# Anti-Inflammatory Effect of Erinacine C on NO Production Through Down-Regulation of NF-κB and Activation of Nrf2-Mediated HO-1 in BV2 Microglial Cells Treated with LPS

**DOI:** 10.3390/molecules24183317

**Published:** 2019-09-12

**Authors:** Li-Yu Wang, Chin-Shiu Huang, Yu-Hsuan Chen, Chin-Chu Chen, Chien-Chih Chen, Cheng-Hung Chuang

**Affiliations:** 1 Department of Nutrition, Master Program of Biomedical Nutrition, Hungkuang University, Taichung City 43302, Taiwan; u99c206@gmail.com; 2 Department of Food Nutrition and Health Biotechnology, Asia University, Taichung City 41354, Taiwan; cshuang@asia.edu.tw; 3 Department of Medical Laboratory Science and Technology, Central Taiwan University of Science and Technology, Taichung City 40601, Taiwan; yhchen2@ctust.edu.tw; 4 Grape King Bio Ltd., Taoyuan City 324, Taiwan; gkbioeng@grapeking.com.tw; 5 Department of Food Science, Nutrition, and Nutraceutical Biotechnology, Shih Chien University, Taipei City 104, Taiwan; 6 Institute of Food Science and Technology, National Taiwan University, Taipei City 10617, Taiwan; 7 Department of Bioscience Technology, Chung Yuan Christian University, Taoyuan City 32023, Taiwan; 8 Department of Cosmetic Science, Chang Gung University of Science and Technology, Taoyuan City 33303, Taiwan; chen37426972@gmail.com

**Keywords:** neuroinflammation, microglial cells, nitric oxide, erinacine C, proinflammatory cytokines, *Hericium erinaceus* mycelium

## Abstract

Previous studies have revealed the anti-inflammatory and neuroprotective properties of *Hericium erinaceus* extracts, including the fact that the active ingredient erinacine C (EC) can induce the synthesis of nerve growth factor. However, there is limited research on the use and mechanisms of action of EC in treating neuroinflammation. Hence, in this study, the inflammatory responses of human BV2 microglial cells induced by LPS were used to establish a model to assess the anti-neuroinflammatory efficacy of EC and to clarify its possible mechanisms of action. The results showed that EC was able to reduce the levels of nitric oxide (NO), interleukin-6 (IL-6), tumor necrosis factor (TNF)-α, and inducible nitric oxide synthase (iNOS) proteins produced by LPS-induced BV2 cells, in addition to inhibiting the expression of NF-κB and phosphorylation of IκBα (p-IκBα) proteins. Moreover, EC was found to inhibit the Kelch-like ECH-associated protein 1 (Keap1) protein, and to enhance the nuclear transcription factor erythroid 2-related factor (Nrf2) and the expression of the heme oxygenase-1 (HO-1) protein. Taken together, these data suggest that the mechanism of action of EC involves the inhibition of IκB, p-IκBα, and iNOS expressions and the activation of the Nrf2/HO-1 pathway.

## 1. Introduction

The occurrence of neurodegenerative diseases such as multiple sclerosis, Parkinson’s disease, and Alzheimer’s disease (AD) is closely related to neuroinflammation [1,2,3]. Neuroinflammation is a chronic, central nervous system (CNS)-specific, and inflammation-like glial response that leads to plaque formation, dystrophic neurite growth, excessive tau phosphorylation, and, ultimately, neurodegenerative diseases [1,3]. Microglial cells are resident macrophages in the CNS, and play an important role in regulating immune responses in the brain [4,5,6]. The abnormal activation of microglial cells is one of the factors that cause neuroinflammation. In particular, phagocytic and cytotoxic functions of microglial cells are triggered by CNS injury [7,8]. The invasion of xenobiotics, such as lipopolysaccharide (LPS) and β-amyloid peptide (Aβ), in the brain often triggers the activation of microglial cells. When activated, microglial cells have phagocytic abilities that allow them to move to the infected region, clean up debris, and produce neurotoxic molecules, such as nitric oxide (NO), and proinflammatory cytokines, such as interleukin (IL)-1β, IL-6, and tumor necrosis factor (TNF)-α, that promote neuroinflammation [5,9,10,11]. In addition, the cytotoxic functions of microglial cells are induced by the release of superoxide radicals and NO into the microenvironment in response to pathogens and cytokine stimulation [12]. Hence, neuroinflammation and neurodegenerative diseases may be prevented by inhibiting the production of neurotoxic chemokines and cytokines by microglial cells [2].

*Hericium erinaceus* (*H. erinaceus*) is often consumed and taken as a health supplement in Taiwan, China, and Japan. In Chinese folk medicine, *H. erinaceus* is used to treat tumors of the digestive system such as esophageal cancer, gastric cancer and duodenal cancer [13,14]. According to various studies, extracts from the fruiting body and mycelium of *H. erinaceus* provide many health benefits, such as anti-oxidizing properties [15,16], anti-inflammatory properties [17,18], the promotion of neuron growth and regeneration [19,20,21], the prevention of memory loss [22,23], and the activation of other physiological functions. For example, recent studies have shown that 4-chloro-3,5-dimethoxybenzoic methyl ester and erincine A isolated from *H. erinaceus* enhance NGF-induced neurite outgrowth and protect neuronally-differentiated cells against deprivation of NGF in PC12 pheochromocytoma cells [21]. Another interesting study suggested that *H. erinaceus* may exert anti-inflammatory effects on macrophages by inhibiting Toll-like receptor (TLR) 4/c-Jun N-terminal kinases (JNKs) signaling and improve adipose tissue inflammation associated with obesity [18]. These health benefits are often linked to cyathane-type diterpenoids, such as erinacine compounds, often found in *H. erinaceus* [24,25,26,27].

**Figure 1:**
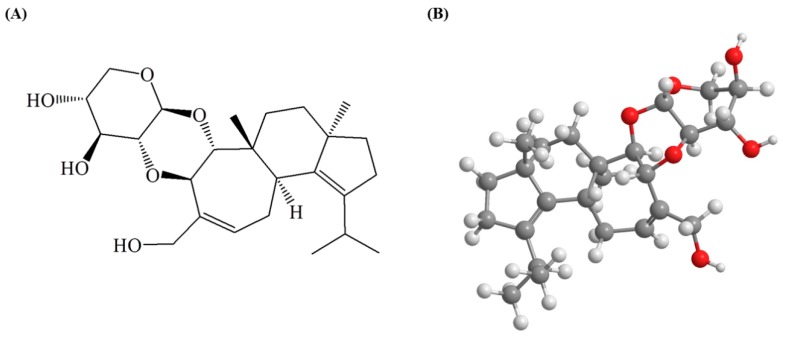
The chemical structure of erinacine C: (**A**) 2-dimensional structure; (**B**) 3-dimensional structure.

Erinacine C (EC, Figure 1) is a secondary metabolite of the mycelium of *H. erinaceus* [28]. In vitro tests revealed that erinacine compounds promote the nerve growth factor (NGF) synthesis of astrocytes in rodents [24,25,29]. For instance, Kawagishi et al. [24] discovered that EC stimulates the production of NGF by astroglial cells in mice, and its stimulatory effect was better than that of epinephrine, a previously known NGF stimulant. Therefore, EC is often regarded to have high potential for treating neurodegenerative diseases such as AD [29]. Furthermore, in a study conducted by Tzeng et al. [27], five-month old APP/PS1 mice were fed 300 mg/kg/day of erinacine A, which significantly reduced the expression of fibrillary acidic protein (GFAP), ionized calcium binding adaptor molecule 1 (Iba1), and Aβ protein in their hippocampuses, in addition to improving their activities of daily living. Chen et al. [26] also discovered that the Aβ plaque loads in the brains of APP/PS1 mice were lowered after the mice were continuously fed with 30 mg/kg/day of erinacine S for 30 days. However, many studies have only indicated that EC can induce the synthesis of NGF, while there has been a lack of reports regarding the role of EC in preventing neuroinflammation and its mechanism of action. Therefore, in this research, the inflammatory responses of LPS-induced BV2 microglial cells were used to establish a model to investigate the anti-neuroinflammatory efficacy of EC and its possible mechanisms of action.

## 2. Results

### 2.1. Effects of EC on Cell Viability

**Figure 2:**
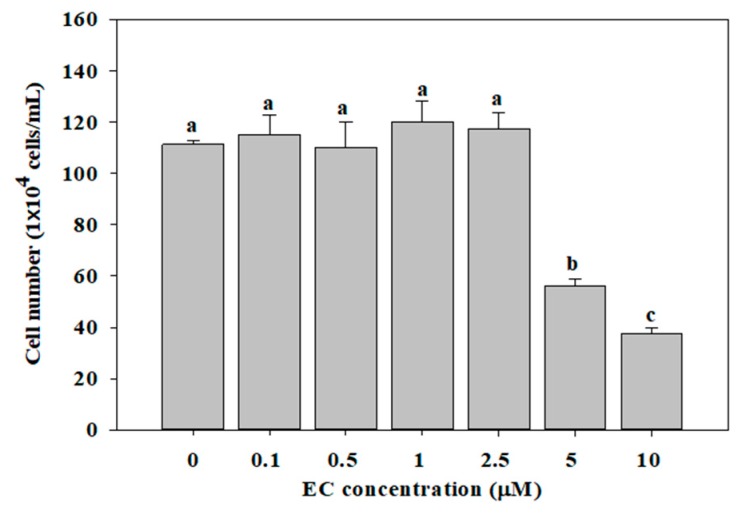
Effects of erinacine C (EC) on cell growth in BV2 microglial cells. Cells were treated with different concentrations of EC (0.1–10 μM) for 24 h. Cell numbers were counted using a hemocytometer. Values (means ± SD, n = 3) not sharing a common lower case letter are significantly different (*p* < 0.05).

To examine the effect of EC on cell viability, we counted the number of treated BV2 cells. As shown in Figure 2, the incubation of BV2 cells with 0.1–2.5 µM EC for 24 h did not decrease cell viability within the range of concentrations used. However, the cell viability was significantly decreased at concentrations of 5 and 10 µM EC. Therefore, we used cells cultured in 0.1–2.5 uM EC for 24 h for the follow-up experiments.

### 2.2. Effects of EC on LPS-Induced Production of NO, IL-6, and TNF-α

**Figure 3:**
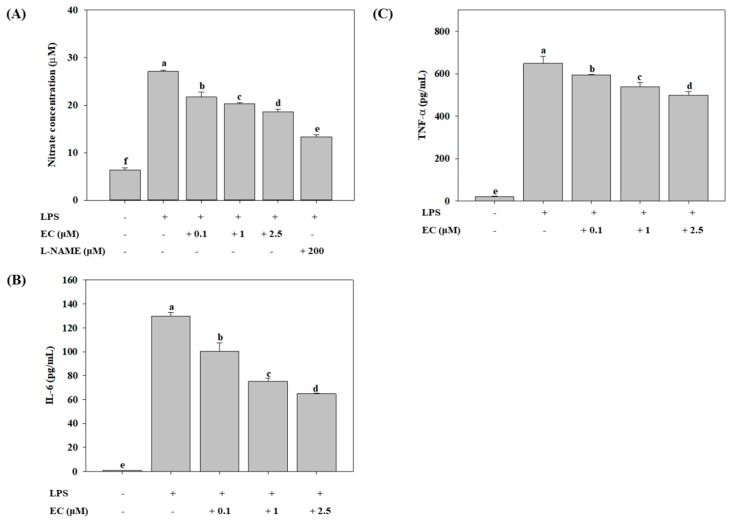
Effects of erinacine C (EC) on LPS-induced production of nitric oxide (**A**; NO), interleukin 6 (**B**; IL-6), and tumor necrosis factor alpha (**C**; TNF-α) in BV2 microglial cells. Cells (1 × 10^5^ cells/mL) were pretreated with different concentrations (0.1–2.5 μM) of EC for 1 h, after which the cells were treated with LPS (500 ng/mL) for 24 h. Culture supernatants were collected and the production of NO, IL-6, and TNF-α was determined by an ELISA kit. l-NAME (200 µM) was used as the positive control. Values (means ± SD, n = 3) not sharing a common lower case letter are significantly different (*p* < 0.05).

LPS is one of the immunostimulatory molecules that can activate inflammation and the production of NO in microglial cells [10,11]. As shown in Figure 3A, LPS induced a significant increase of NO levels in BV2 cells. EC significantly inhibited the LPS-induced production of NO in a dose-dependent manner, with a maximum inhibition of 31% at 2.5 µM EC, compared to the LPS-treated groups (*p* < 0.05). N(G)-Nitro-l-arginine methyl ester (l-NAME) was used as the positive control, and the addition of 200 µM of l-NAME significantly inhibited the LPS-induced production of NO by 51% (*p* < 0.05). In addition, activated microglial cells are a major source of inflammatory cytokines such as IL-6 and TNF-α, both of which can be up-regulated in various inflammatory diseases [5,11]. We analyzed the production of IL-6 and TNF-α induced by LPS using an ELISA kit. As shown in Figure 3B,C, the levels of IL-6 and TNF-α were significantly increased in BV2 cells treated with LPS (*p* < 0.05). However, EC significantly inhibited LPS-induced levels of IL-6 and TNF-α in a concentration-dependent manner. When EC was added at 2.5 µM, the levels of IL-6 and TNF-α were reduced to 50% (*p* < 0.05) and 23% (*p* < 0.05), respectively, as compared with those of LPS-treated group.

### 2.3. Effects of EC on LPS-Induced Protein Expression of iNOS

**Figure 4:**
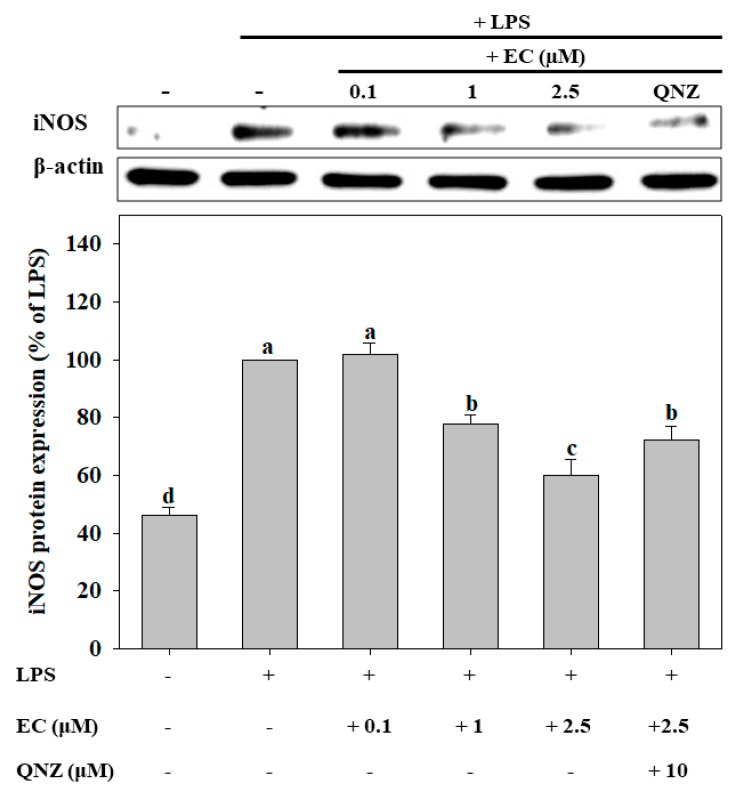
Effects of erinacine C (EC) on LPS-induced iNOS expression in BV2 microglial cells. Cells (1 × 10^5^ cells/mL) were pretreated with different concentrations (0.1–2.5 μM) of EC for 1 h, after which the cells were treated with LPS (500 ng/mL) for 24 h. iNOS protein expression was determined by western blot analyses. Values (means ± SD, n = 3) not sharing a common lower case letter are significantly different (*p* < 0.05). The western blot image is provided as a representative result of multiple experiments.

The protein expression of inducible nitric oxide synthase (iNOS) in BV2 cells was determined by western blot. As shown in Figure 4, EC inhibited the iNOS protein expression induced by LPS in a dose-dependent manner. Defining the iNOS protein expression in the LPS-treated group as 100% expression in order to quantify such expression in other groups, it can be seen that iNOS protein expression was significantly inhibited by 40% in cells treated with 2.5 μM EC (*p* < 0.05). We further demonstrated that 4-*N-*[2-(4-phenoxyphenyl) ethyl]quinazoline-4,6-diamine (QNZ), an inhibitor of nuclear factor kappa-light-chain-enhancer of activated B cells (NF-κB), at 10 μM, significantly suppressed the inhibitory effect of 2.5 μM EC on the expression of iNOS in BV2 cells (*p* < 0.05).

### 2.4. Effects of EC on LPS-Induced NF-κB, p-IκBα, and IκBα Protein Expression in BV2 cells

**Figure 5:**
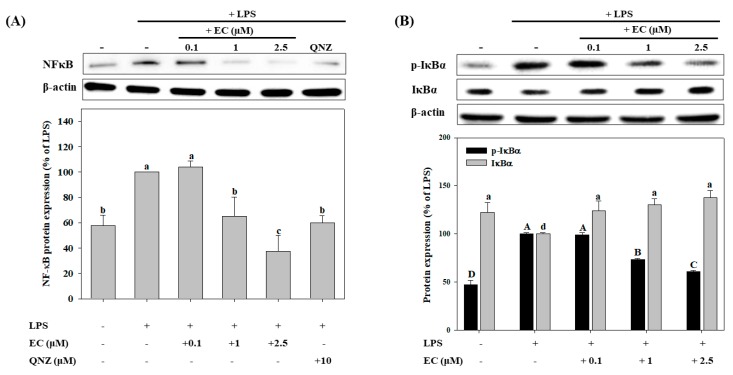
Effects of erinacine C (EC) on LPS-induced NFκB (**A**), p-IκBα, and IκBα (**B**) protein expression in BV2 microglial cells. Cells were pretreated with different concentrations (0.1–2.5 μM) of EC for 1 h, after which the cells were treated with LPS (500 ng/mL) for 24 h. NFκB, p-IκBα, and IκBα protein expressions were determined by western blot analyses. Values (means ± SD, n = 3) not sharing a common capital (or a lower case letter) are significantly different (*p* < 0.05). The western blot image is provided as a representative result of multiple experiments.

NF-κB activation is an important mediator the LPS-induced expression of iNOS in BV2 cells [30,31]. We further determined whether EC suppressed the activation of NF-κB by measuring the LPS-induced phosphorylation of IκBα (p-IκBα) in BV2 cells using western blot analysis. As shown in Figure 5, LPS significantly up-regulated the expression of the NF-κB and p-IκBα proteins. However, at 1 µM and 2.5 µM, EC significantly decreased the protein expression of NF-κB and p-IκBα in a concentration-dependent manner. The 2.5 µM EC significantly decreased the protein expression of NF-κB and p-IκBα by 70% and 39%, respectively, while QNZ decreased the expression of NF-κB by 51%, as compared with that of the LPS-treated group (*p* < 0.05).

### 2.5. Effects of EC on Nrf2 and HO-1 Protein Expression in BV2 cells

**Figure 6:**
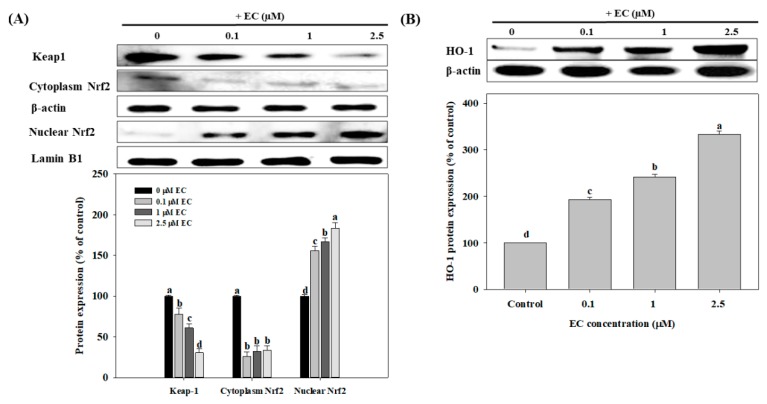
Effects of erinacine C (EC) on Nrf2, Keap-1 (**A**), and HO-1 (**B**) protein expression in BV2 microglial cells. Cells were treated with different concentrations (0.1–2.5 μM) of EC for 24 h. Nrf2, Keap-1, and HO-1 protein expressions were determined by western blot analyses. Values (means ± SD, n = 3) not sharing a common lower case letter are significantly different (*p* < 0.05). The western blot image is provided as a representative result of multiple experiments.

Nuclear transcription factor erythroid 2-related factor (Nrf2) is a transcription factor that regulates the coordinated expression of heme oxygenase-1 (HO-1). Nrf2 resides in the cytoplasm by forming an inactive complex with an Nrf2-inhibitory protein called Kelch-like ECH-associated protein 1 (Keap1) [30]. Thus, we next evaluated the expression of Keap1, Nrf2, and HO-1 proteins in BV2 cells. As shown in Figure 6A, EC significantly up-regulated the expression of nuclear Nrf2 protein in a dose-dependent manner (*p* < 0.05). Compared to the control group, EC at 0.1, 1, and 2.5 µM significantly increased the protein expression of nuclear Nrf2 by 56%, 67%, and 83%, respectively (*p* < 0.05). Meanwhile, although EC significantly decreased the protein expression of Nrf2 in the cytoplasm compared to the control group, the differences among the groups were not significant (*p* > 0.05). As shown in Figure 6B, EC significantly increased HO-1 protein expression in a dose-dependent manner (*p* < 0.05). Compared to the control group, the addition of EC at 0.1, 1, and 2.5 µM significantly increased the protein expression of HO-1 by 93%, 141%, and 233%, respectively (*p* < 0.05).

## 3. Discussion

The occurrence of neurodegenerative diseases is closely related to neuroinflammation. Studies have shown that the activation of microglial cells can be induced by LPS, which produces large amounts of reactive oxygen species (ROS), NO, and proinflammatory cytokines such as IL-6 and TNF-α that damage neurons and ultimately cause neurodegenerative diseases [3,5,11]. In this research, EC, which is found in extracts from the mycelium of *H. erinaceus*, reduced the NO, IL-6, and TNF-α produced by LPS-induced BV2 cells. The activation of iNOS in cells can be stimulated by LPS, TNF-α, or IL-1 [9]. The activated iNOS protein promotes the conversion of l-arginine to L-citrulline in cells, which in turn, produces and releases large amounts of NO [31,32,33]. Moreover, in this research, EC was found to inhibit the expression of the iNOS protein, as a statistically significant difference was observed (*p* < 0.05). Therefore, it is suggested that the reduced expression of the iNOS protein may be related to the ability of EC to inhibit LPS-induced NO.

LPS induces the generation of various ROS, which triggers neuroinflammation and modulates the redox-sensitive signal transduction pathways and transcription factors in cells [10,11]. The NF-κB plays a key role in redox-sensitive signal transduction pathways. According to Karki et al. [34], QNZ is a specific inhibitor of NF-κB. In this research, BV2 cells were cultivated in a mixture of QNZ and EC. The results indicated that QNZ weakened the inhibitory effect of EC on iNOS expression, suggesting that EC exerts its effect through modulating the NF-κB signaling transduction pathway.

Under normal physiological conditions, NF-κB is sequestered in the cytoplasm by its inhibitory protein, IκBα. When IκBα becomes phosphorylated, it undergoes ubiquitination and degradation, which results in the release and nuclear translocation of NF-κB, inducing the expression of specific target genes, such as TNF-α, IL-6, and iNOS [3,11,35]. Previous studies have shown that the expression of NF-κB in the nucleus was increased when BV2 cells were stimulated by LPS, resulting in the activation of iNOS and production of NO [33,35,36]. The results of this research were consistent with those in the aforementioned literature. Furthermore, a dose-response relationship was observed, and EC was found to significantly inhibit the expressions of the NF-κB and p-IκBα proteins. Therefore, it is suggested that EC lowers concentrations of LPS-induced NO, IL-6, and TNF-α, and inhibits the expression of iNOS, by down-regulating the expression of the NF-κB and p-IκBα proteins.

Another possible mechanism was suggested in the literature, that is, the activation of the Nrf2/HO-1 signaling pathway reduces LPS-induced cellular damage and inflammatory responses [30,37,38]. As an inducible isoform, the expression of HO-1 is up-regulated by various oxidants [30,39]. HO-1 is regarded to have modulating, immune-regulating, and anti-inflammatory functions [30,37,38,39]. Furthermore, the Nrf2 is sequestered in the cytoplasm by its inhibitory protein, Keap1. When the cell is stimulated by oxidants, Nrf2 is released and induced by Keap1, which causes Nrf2 to undergo nuclear translocation and interact with the antioxidant response elements (AREs) of HO-1 [40]. In this research, a dose-response relationship was observed, and EC was found to significantly inhibit the expression of the Keap1 protein and increase the expression of the Nrf2 protein in the nucleus. Hence, it is suggested that certain mechanisms of the anti-inflammatory responses induced by EC were achieved by up-regulating the Nrf2/HO-1 signaling pathway.

## 4. Materials and Methods

### 4.1. Chemicals and Reagents

The EC (purity > 98%, molecular formula C_25_H_38_O_6_, molecular weight 434) was a gift from Dr. Chien-Chih Chen (Chang Gung University of Science and Technology, Taoyuan city, Taiwan). Its structure was confirmed based on its ^13^C- and ^1^H-NMR spectra (Supplementary Figure S1 [24]). LPS from *Escherichia coli* serotype O55:B5 (L6529), ammonium persulfate (APS), Trizma base (Tris), and bromophenol blue were purchased from Sigma-Aldrich (St. Louis, MO, USA). Fetal bovine serum (FBS), trypsin, DMEM, and penicillin/streptomycin were purchased from Gibco/BRL (Rockville, MD, USA). QNZ (EVP4593) was purchased from Selleckchem (Houston, TX, USA). NF-κB monoclonal antibody (mAb; cat. no. sc-8008), p-IκBα mAb (cat. no. sc-8404), Keap-1 mAb (cat. no. sc-33569), Nrf2 mAb (cat. no. sc-365949), HO-1 mAb (cat. no. sc-390991), and Lamin B1 mAb (cat. no. sc-37700) were purchased from Santa Cruz (Dallas, TX, USA). iNOS mAb (cat. no. bs-2072R) was purchased from Bioss Antibodies (Woburn, MA, USA). IκBα mAb (cat. no. MS5-15132) was purchased from Thermo Fisher Scientific (Waltham, MA, USA). The above reagents were of reagent grade I or molecular biology grade.

### 4.2. Extraction and Isolation of EC

The mycelia of *H. erinaceum* were refluxed with 95% ethanol. The ethanol solution was concentrated in vacuum to give a brown extract which was partitioned with H_2_O/EtOAc (1:1) to afford a H_2_O layer and an EtOAc layer. The EtOAc layer was chromatographed on a silica gel column (70–230 mesh, 70 × 10 cm), eluting with a gradient system of *n*-hexane/EtOAc (10:1; 3:1; 3:2; 1:1; 1:2; 0:1) to give seven fractions (Fr. I−VII). Fraction VI, the eluate of *n*-hexane/EtOAc (1:2), was separated on a Sephadex LH-20 column eluting with MeOH to yield two sub-fractions (Fr. VI-1 and VI-2). Sub-fraction VI-1 was further purified with Sephadex LH-20 column eluted with MeOH to give erinacine C.

### 4.3. Cell Culture

Cell culture was performed as described previously [2,41]. Briefly, the BV2 microglial cells (a gift from Dr. Yuh-Chiang Shen, National Research Institute of Chinese Medicine, Taipei, Taiwan) were cultured in DMEM medium containing 10% (*v*/*v*) fetal bovine serum (FBS), 0.37% (*w*/*v*) NaHCO_3_, penicillin (100 units/mL), and streptomycin (100 μg/mL) at 37 °C in a humidified incubator under 5% CO_2_ and 95% air. Equal numbers of cells (1 × 10^4^/mL) were incubated for 24 h before being subjected to the various treatments. Before the experiment, the medium was removed, and the cells were washed twice with phosphate-buffered saline (PBS). Then, new media (with 10% FBS) containing various concentrations (0.1–10 μM) of EC were added, and the samples were incubated for 1 h, after which the cells were treated with LPS (0.5 µg/mL) for 24 h. In addition, the effects of l-NAME (200 µM) and QNZ (20 μM) were also evaluated, with the cells treated with them used as a positive control. Stock solutions of EC, QNZ, and l-NAME were dissolved in DMSO. Before being used, the compounds were diluted in 10% FBS in culture medium to the desired concentrations at the time of addition. The highest concentration of DMSO used did not exceed 0.1% (*v*:*v*) of the total assay volume, which did not affect cell viability.

### 4.4. Cell Growth Analysis

The cell growth was assayed as described in a previous study by Chuang et al. [41]. BV2 cells were plated in 24-well plates at a density of 1 × 10^5^ cells/well and grown for 24 h. Different concentrations of EC were then added to the cells to reach final concentrations of 0.1, 0.5, 1, 2.5, 5, and 10 µM in the presence of FBS. The control group only contained 10% FBS. The cells were then grown at 37 °C, 5% CO_2_, and 95% air for different periods of time, and the trypan blue exclusion protocol was used to determine cell viability.

### 4.5. Measurement of NO, TNF-α, and IL-6

Measurements of NO, TNF-α, and IL-6 were performed as described by Santhosh et al. [42] and Barberi et al. [43], with minor modifications. BV2 cells were incubated with various concentrations of EC for 1 h prior to incubation with or without LPS (0.5 µg/mL) for a further 24 h. The total NO concentration in culture supernatants was estimated using a colorimetric assay kit (cat. no. 780001; Cayman Chemicals, Ann Arbor, MI, USA). TNF-α and IL-6 concentrations in culture supernatants were assessed using the commercial cytokine ELISA kit (cat. no. 431301 and 430901; BioLegend, San Diego, CA, USA), according to the manufacturer’s instructions.

### 4.6. Western Blotting

Expression levels of NF-κB, IκB, p-IκBα, Keap-1, Nrf2, HO-1, and iNOS proteins were determined by western blotting. Western blot analysis was performed as described previously [41]. Briefly, the medium was removed and cells were lysed with 20% sodium dodecyl sulphate (SDS) containing 1 mM phenylmethylsulfonyl fluoride. The lysate was sonicated for 30 s on ice followed by centrifugation at 12,000× *g* for 30 min at 4 °C. An amount of protein (40 μg) from the supernatant was resolved by SDS-polyacrylamide gel electrophoresis (PAGE) and transferred onto a nitrocellulose membrane. After blocking with Tris-buffered saline buffer (20 mM Tris-HCl, 150 mM NaCl, pH 7.4) containing 5% nonfat milk, the membrane was incubated with anti-NF-κB mAb, anti-IκBα mAb, anti-p-IκBα mAb, anti-Keap-1 mAb, anti-Nrf2 mAb, anti-HO-1 mAb, and anti-iNOS mAb followed by horseradish peroxidase-conjugated anti-mouse IgG and then visualized using an ECL chemiluminescent detection kit (Millipore, Billerica, MA, USA). The relative levels of NF-κB, IκB, p-IκBα, Keap-1, Nrf2, HO-1, and iNOS proteins were quantitated using Matrox Inspector version 2.1 software (Matrox, Dorval, QC, Canada).

### 4.7. Statistical Analysis

Statistical analysis was performed as described previously [41]. Values were expressed as means ± SD and analyzed using one-way ANOVA, followed by the application of Duncan’s Multiple Range Test for comparisons of group means. The statistical analysis was performed using SPSS version 10 (IBM Inc., Armonk, NY, USA. *P* values < 0.05 were considered statistically significant.

## 5. Conclusions

In summary, we have shown here that EC reduced the concentrations of LPS-induced pro-inflammatory factors such as IL-6, TNF-α, and NO. It is postulated that the mechanism of action of EC involves the inhibition of the expressions of NF-κB, p-IκBα, and iNOS. EC also activates the Nrf2 signaling pathway, which increases the expression of HO-1. More studies are required to further elucidate the anti-inflammatory mechanisms of EC.

## Supplementary Materials

The following are available online. Figure S1: ^13^C-NMR spectrum (A) and ^1^H-NMR spectrum (B) of Erinacine C.

## Abbreviations

4-*N*-[2-(4-Phenoxyphenyl)ethyl]quinazoline-4,6-diamine, QNZ; nitric oxide, NO; lipopolysaccharide, LPS; β-amyloid peptide, Aβ; interleukin, IL; erinacine C, EC; nuclear transcription factor erythroid 2-related factor, Nrf2; Kelch-like ECH-associated protein 1, Keap1; antioxidant response elements, AREs; nuclear factor kappa-light-chain-enhancer of activated B cells, NF-κB; inducible nitric oxide synthase, iNOS; reactive oxygen species, ROS; Alzheimer’s disease, AD; N(G)-nitro-l-arginine methyl ester, l-NAME.
