# Supplementary material for: Anti-Inflammatory Effect of Erinacine C on NO Production Through Down-Regulation of NF-κB and Activation of Nrf2-Mediated HO-1 in BV2 Microglial Cells Treated with LPS
Source: Molecules. 2019 Sep 12;24(18):3317. doi: 10.3390/molecules24183317 (PMC6766924; doi:10.3390/molecules24183317)
Supplement: Supplementary file 1 [file molecules-24-03317-s001.pdf]

(A)

(B)

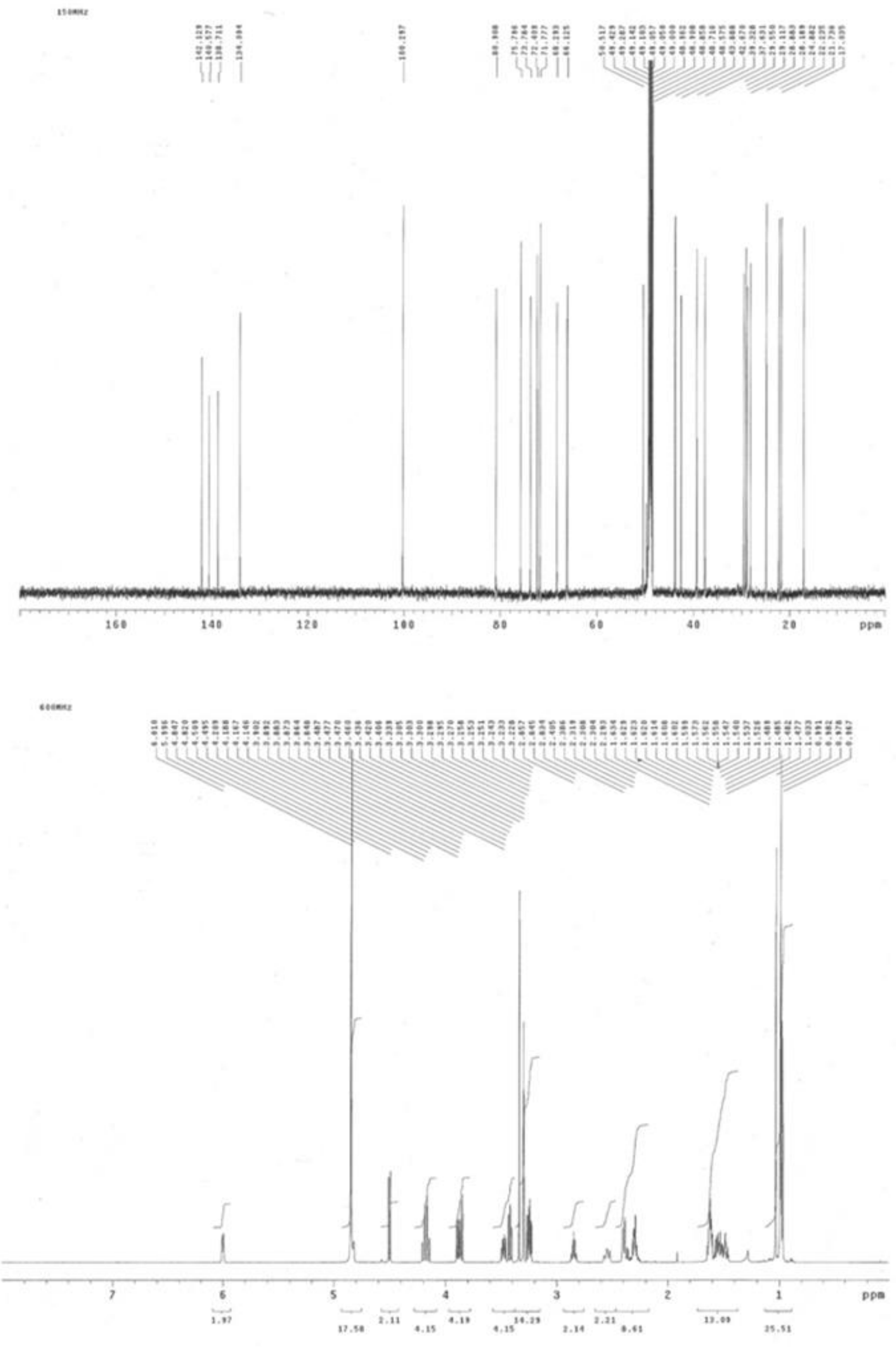

Supplementary figure 1. ^{13}C-NMR spectrum (A) and ^{1}H-NMR spectrum (B) of Erinacine C
